# FastICA peel-off for ECG interference removal from surface EMG

**DOI:** 10.1186/s12938-016-0196-8

**Published:** 2016-06-13

**Authors:** Maoqi Chen, Xu Zhang, Xiang Chen, Mingxing Zhu, Guanglin Li, Ping Zhou

**Affiliations:** Department of Electronic Science and Technology, University of Science and Technology of China, Hefei, China; Guangdong Provincial Work Injury Rehabilitation Center, Guangzhou, China; The Key Laboratory of Human-Machine Intelligence-Synergy Systems, Shenzhen Institutes of Advanced Technology, Chinese Academy of Sciences, Shenzhen, China; Department of Physical Medicine and Rehabilitation, University of Texas Health Science Center and TIRR Memorial Hermann Research Center, Houston, USA

**Keywords:** Independent component analysis, Multi-channel EMG recording, ECG interference elimination

## Abstract

**Background:**

Multi-channel recording of surface electromyographyic (EMG) signals is very likely to be contaminated by electrocardiographic (ECG) interference, specifically when the surface electrode is placed on muscles close to the heart.

**Methods:**

A novel fast independent component analysis (FastICA) based peel-off method is presented to remove ECG interference contaminating multi-channel surface EMG signals. Although demonstrating spatial variability in waveform shape, the ECG interference in different channels shares the same firing instants. Utilizing the firing information estimated from FastICA, ECG interference can be separated from surface EMG by a “peel off” processing. The performance of the method was quantified with synthetic signals by combining a series of experimentally recorded “clean” surface EMG and “pure” ECG interference.

**Results:**

It was demonstrated that the new method can remove ECG interference efficiently with little distortion to surface EMG amplitude and frequency. The proposed method was also validated using experimental surface EMG signals contaminated by ECG interference.

**Conclusions:**

The proposed FastICA peel-off method can be used as a new and practical solution to eliminating ECG interference from multichannel EMG recordings.

## Background

Electromyography (EMG) signal provides important information on muscle physiology and function, and has a variety of applications. When surface electrode is placed on muscles close to the heart (such as trunk muscles), the recorded EMG signals are contaminated by electrocardiography (ECG) interference. The ECG interference overlaps with EMG in both time domain and frequency domain, making it difficult to be removed with conventional filters. To remove ECG interference from EMG signals, a high-pass filter [1, 2] is often used with a cutoff frequency of approximately 30 Hz to 60 Hz, or even higher. Such a setting will unavoidably distort useful EMG signal components due to the frequency overlapping. Other more complicated methods have also been developed including ECG template subtraction, wavelet thresholding, adaptive filtering, etc. [3–7] With the development of blind source separation techniques, independent component analysis (ICA) has been used for ECG interference removal from surface EMG or other bioelectrical signals [8, 9]. A widely used method is the well-known FastICA, which can be combined with other techniques (such as adaptive filter and wavelet analysis [10, 11]) for ECG artifact removal. Most of the ICA studies viewed ECG interference as an independent component instantaneously superimposed with the EMG signal. The ICA was usually applied to extract the ECG component from the mixed signals, which can be used in subsequent processing for the purpose of noise removal. By treating the contaminated signals as an instantaneous mixing model, the ECG waveform variation across different channels was not considered. In this study, we proposed a more complex and realistic shift invariant convolutive data mixing model to describe ECG contaminated EMG signals [12]. Based on such a model, a novel FastICA peel-off technique was developed to remove the ECG interference from multi-channel surface EMG signals. The developed method is designed to allow precise estimation and subtraction of ECG waveforms for individual channels, thus enabling improved ECG interference removal with little distortion to the useful EMG signals. The performance of the novel technique was validated by both synthetic and experimental approaches.

## Methods

### Data model

An ECG contaminated multi-channel surface EMG signal can be described by the shift-invariant convolutive model. For a specific channel *i*,

1 $$ EMG_{i}^{C} \left( t \right) = EMG_{i} \left( t \right) + ECG_{i} \left( t \right) + \omega_{i} \left( t \right);\quad i = 1, \ldots M $$

where $EMG_{i}^{C}$ represents the *i*th channel surface EMG signal contaminated by ECG interference; *EMG*_*i*_ represents the clean EMG signal; *ECG*_*i*_ represents the ECG interference; and *ω*_*i*_ represents all the other noise in addition to the ECG interference. *M* is the number of recording channels. In more details, we can represent the ECG interference in the form of convolution:

2 $$ ECG_{i} \left( t \right) = \mathop \sum_{\tau = 0}^{L - 1} A_{i} \left( \tau \right)S\left( {t - \tau } \right) $$

where *A*_*i*_ is the vector denoting ECG waveform in the *i*th channel, and *L* represents the length of the waveform. *S* is a binary pulse sequence (i.e. either 0 or 1) along the time line, where “1” indicates an occurrence of the ECG spike. In this study we focus on how to separate ECG interference from surface EMG, more information about nature of the EMG signal (i.e. *EMG*_*i*_) in terms of different motor unit action potential trains can be found in literature [1–4, 6–8, 12].

### FastICA

FastICA is one of the most popular and effective methods for blind source separation [13]. For the pre-whitened signal **x**, to find one independent component *y* = **w**^*T*^**x**, the following optimization problem needs to be solved:

3 $$ \begin{aligned} \hbox{max} \quad J_{G} \left( {\mathbf{w}} \right) = \left[ {E\left\{ {G\left( {{\mathbf{w}}^{T} {\mathbf{x}}} \right)} \right\} - E\left\{ {G\left( \upsilon \right)} \right\}} \right]^{2} \\ s.t.\quad h\left( {\mathbf{w}} \right) = E\left\{ {y^{2} } \right\} - 1 = \left\| {\mathbf{w}} \right\|_{2}^{2} - 1 = 0 \end{aligned} $$

where *G* is a nonquadratic function [a default use can be *G*(*x*) = log (cosh (*x*))], *υ* is a standard normal random variable. Then an updating rule based on a fixed-point algorithm is presented below [13]:

4 $$ \begin{aligned} {\mathbf{w}}^{ + } &= E\left\{ {{\mathbf{x}}G^{\prime}\left( {{\mathbf{w}}^{T} {\mathbf{x}}} \right)} \right\} - E\left\{ {G^{\prime\prime}\left( {{\mathbf{w}}^{T} {\mathbf{x}}} \right)} \right\}{\mathbf{w}} \\ {\mathbf{w}}^{ * } &={{{\mathbf{w}}^{ + } } \mathord{\left/ {\vphantom {{{\mathbf{w}}^{ + } } {\left\| {{\mathbf{w}}^{ + } } \right\|}}} \right. \kern-0pt} {\left\| {{\mathbf{w}}^{ + } } \right\|}}_{2} \end{aligned} $$

In order to facilitate the application of FastICA and improve the numerical conditioning, the data model (1) can be extended in the channel direction by *K* − 1 delayed repetitions of each observation [12]:

5 $$ \overline{{EMG^{C} }} \left( t \right) = \left[ {EMG_{1}^{C} \left( t \right),EMG_{1}^{C} \left( {t - 1} \right), \ldots ,EMG_{1}^{C} \left( {t - K + 1} \right), \ldots ,EMG_{M}^{C} \left( t \right), \ldots ,EMG_{M}^{C} \left( {t - K + 1} \right)} \right]^{T} $$

where the delay factor *K* denotes the total number of time intervals to be delayed. A greater *K* value helps to improve FastICA performance by taking a wider range of time shift in the convolutive model into account, and therefore results in larger burden of computation. Considering this trade-off, the delay factor *K* was determined to be 5 (e.g. 5 ms under a sampling rate of 1 kHz) after some pretests. It was also found in the current study that the FastICA performance was insensitive to a slight variation of *K*. After applying FastICA on $\overline{{EMG^{C} }}$, a rough estimate of *S* can be extracted through a thresholding method applied on an appropriate output independent component corresponding to the ECG interference, which simply makes values as 1 at spike occurrences and values as 0 beyond individual spikes. Note that the spike morphology across the independent components (which are just the filtering output derived from different channels along with their delays) does not deliver meaningful information and therefore is ignored.

### Constrained FastICA

In this study, we use signal to interference ratio (SIR) to describe the degree of ECG contamination for a specific channel *i*. The SIR is defined as:

6 $$ {\text{SIR}}\left( i \right) = 10 \cdot { \lg }\left( {\frac{{\mathop \sum \nolimits_{t} EMG_{i}^{2} \left( t \right)}}{{\mathop \sum \nolimits_{t} ECG_{i}^{2} \left( t \right)}}} \right) $$

It is obvious that the lower SIR of the signal, the easier FastICA can estimate the ECG spike train precisely. In some cases, especially when the SIR is high, false or missing spikes may take place in the estimated spike train. We propose the use of constraint FastICA [14] to further assess and validate the result. More specifically, we use the rough estimate of the ECG spike train (i.e. *S*) identified from the initial FastICA output as a constraint to process the contaminated signal again using FastICA. Such a constraint is able to drive FastICA to converge toward an independent component mostly similar to the estimated spike train, so the output of constrained FastICA can be used to correct possible false or missing spikes from the initial FastICA processing. This process allows *S* to be updated, and can be repeated until reaching a convergence.

Compared to FastICA, the optimization problem of the constrained FastICA is described below. For the pre-whitened data **x**,

7 $$ \begin{aligned} \hbox{max} \quad J_{G} \left( {\mathbf{w}} \right) &= \left[ {E\left\{ {G\left( {{\mathbf{w}}^{T} {\mathbf{x}}} \right)} \right\} - E\left\{ {G\left( \upsilon \right)} \right\}} \right]^{2} \hfill \\ s.t.\quad g\left( y \right) &= \xi - E\left\{ {y^{T} S} \right\} \le 0 \hfill \\ \quad \quad h\left( {\mathbf{w}} \right) &= E\left\{ {\left( {{\mathbf{w}}^{T} {\mathbf{x}}} \right)^{2} } \right\} - 1 = \left\| {\mathbf{w}} \right\|^{2} - 1 = 0 \hfill \\ \quad \quad E\left\{ {S^{2} } \right\} - 1 &= 0 \hfill \\ \end{aligned} $$

where *g*(*y*) is a measure of closeness between output *y* = **w**^*T*^**x** and reference ECG spike train *S* in the sense of correlation. *ξ* (0 ≤ *ξ* ≤ 1) is a preset lower bound of the optimum correlation.

The updating rule of **w** is presented as [14]:

8 $$ \begin{aligned} {\mathbf{w}}^{ + } = E\left\{ {{\mathbf{x}}G^{\prime}\left( {{\mathbf{w}}^{T} {\mathbf{x}}} \right)} \right\} - \mu E\left\{ {{\mathbf{x}}g^{\prime}\left( y \right)} \right\} \hfill \\ {\mathbf{w}}^{ * } = {{{\mathbf{w}}^{ + } } \mathord{\left/ {\vphantom {{{\mathbf{w}}^{ + } } {\left\| {{\mathbf{w}}^{ + } } \right\|}}} \right. \kern-0pt} {\left\| {{\mathbf{w}}^{ + } } \right\|}}_{2} \hfill \\ \mu^{ + } = \hbox{max} \left\{ {0,\mu + \gamma g\left( y \right)} \right\} \hfill \\ \end{aligned} $$

where *μ* and *γ* denote the Lagrange multiplier and the penalty factor introduced in the augmented Lagrangian method, respectively. Please refer to [14] for more details on its implementation. Similar to the FastICA without a constraint, the constrained FastICA can also be applied on the extended form (i.e. $\overline{{EMG^{C} }}$) as described in Eq. (5). As compared with the FastICA, its constrained version requires a relatively larger value of K for improved performance. Therefore, the delay factor *K* used for the constrained FastICA process was set to be 20 in this study.

### ECG interference subtraction

After obtaining a reliable estimation of ECG firing spike train *S*, we utilize this information to estimate the ECG waveform in *i*th channel by solving the following least squares problem:

9 $$ \hbox{min} \left( {X_{i} - A_{i} * S} \right)^{T} \left( {X_{i} - A_{i} * S} \right) $$

where *X*_*i*_ denotes the vector containing all the sample points of $$ EMG_{i}^{C} $$, *A*_*i*_ is the waveform of *ECG*_*i*_ to be estimated, $$ A_{i} * S$$ denotes the convolutive vector containing all the samples as in (2). The analytical solution can be expressed as [12]:

10 $$ A_{i}^{*}  = \left({\overset \frown{S}}^{T}{\overset \frown{S}}\right)^{-1}{\overset \frown{S}}^{T}X_{i}$$

where $\overset\frown{S}$ is a toeplitz matrix formed by all the elements of *S*, which satisfies $\overset\frown{S} A_{i} = A_{i} * S$.

The solution $$ A_{i}^{*}$$ is the least squares estimation of ECG waveforms in the *i*th channel. Given the solution, the surface EMG signal of the *i*th channel can be estimated by subtracting the ECG interference, i.e. $$ X_{i} - \overset{\lower0.5em\hbox{$\smash{\scriptscriptstyle\frown}$}}{S} {\rm A}_{i}^{ * } $$.

### Evaluation data description

Both synthetic and real EMG signals were used to evaluate the performance of the proposed method in ECG interference removal. The synthetic signals were obtained by combining “pure” ECG signals with “clean” surface EMG recordings free of ECG artifacts. In this study we focus on ECG interference and do not concern about other types of noise (e.g. power interference). The signals were recorded by a 128-channel Refa EMG system (TMS International BV, Enschede, Netherlands) at a sampling rate of 1 kHz per channel, with a band pass filter setting at 10–500 Hz. Each channel was recorded using an individual electrode 10 mm in diameter. The dataset used in this study was chosen from recordings of two amputee subjects (one at the transhumeral level, the other at the transradial level, both male, 24 and 25 years, respectively). All the experiment procedures were approved by the institutional ethics committee, and the subjects’ informed consent was given before the experiment. In this study, the following three types of signals were selected and used.

I. “Clean” EMG signals. Such signals were recorded via 128 individual electrodes placed on the left forearm of the transradial amputee (left side) subject. The subject was asked to (or imagine to) stretch his left arm forward, backward, and laterally in three trials, respectively. For each trial, six repetitions of the same movement were performed with a resting period in between. Approximate 1-min data recordings were obtained for each trial. Since the electrode positions were far from the heart, the signals can be viewed free of ECG interference.
II. ECG contaminated EMG signals. The EMG signals with ECG interference were recorded via 128 individual electrodes placed on the left chest and shoulder of the transhumeral amputee (left side) subject. The subject was asked to perform the same three movements as the transradial amputee subject. Since the electrode positions were placed close to the heart, the recorded EMG signals were seriously contaminated by ECG interference.
III. “Pure” ECG signals. With the same electrode position on the transhumeral amputee subject, we also recorded signals when the subject was asked to maintain completely relaxed without any voluntary muscle contraction. These signals were considered as “pure” ECG interference.

By directly mixing each trial of “clean” surface EMG signals and the “pure” ECG signals, we obtained three trials of 128-channel synthetic EMG signals contaminated by ECG interference. These signals were used to quantitatively evaluate the ECG removal performance. To minimize possible power interference, a low pass filter with cutoff frequency of 40 Hz was used to process the “pure” ECG signals. Such a process was not applied to the “clean” EMG signals.

Note that in the FastICA processing, the ECG interference is treated as the target signal to be extracted. It follows that a lower SIR (i.e. ECG interference is relatively more evident in the signal) makes it easier to detect the ECG firing spike train. To further demonstrate the effectiveness of the developed method in high SIRs (with relatively smaller ECG spike appearance), a special dataset consisting of 22 channels in total with SIR larger than 5 dB in each channel were purposely chosen from the synthetic EMG during backward movement for performance evaluation. In these channels the EMG amplitudes are much higher than ECG interferences.

Finally, the developed method was also tested with experimental surface EMG signals contaminated by ECG interference (i.e. II).

### Performance evaluation

In order to evaluate the ECG removal performance of the proposed method, we calculated three indices: the SIR [as defined in Eq. (6), also equivalent to the common signal to noise ratio], correlation coefficient (CORR) and median frequency variation ratio (MFVR) between original “clean” EMG and the contaminated/filtered signals before and after the denoising process, respectively. For the filtered signal, the residual from the “clean” EMG was viewed as the noise/interference in the SIR calculation. The median frequency variation ratio (MFVR) was defined to specifically measure the influence of the interference/residual on the frequency of EMG component:

11 $$ {\text{MFVR}}\left( {\text{i}} \right) = \frac{{\left| {MF_{{EMG_{i} }} - MF_{{EMGC_{i} }} } \right|}}{{MF_{{EMG_{i} }} }} \times 100\,\% $$

where $MF_{{EMG_{i} }}$ denotes the median frequency of the “clean” EMG signal in *i*th channel, and $MF_{{EMGC_{i} }}$ denotes the median frequency of the contaminated EMG signal (before denoising) or the filtered EMG signal (after denoising). Here, median frequency is defined as a frequency at which 50 % of the total power of a signal segment is reached.

## Results

**Fig. 1:**
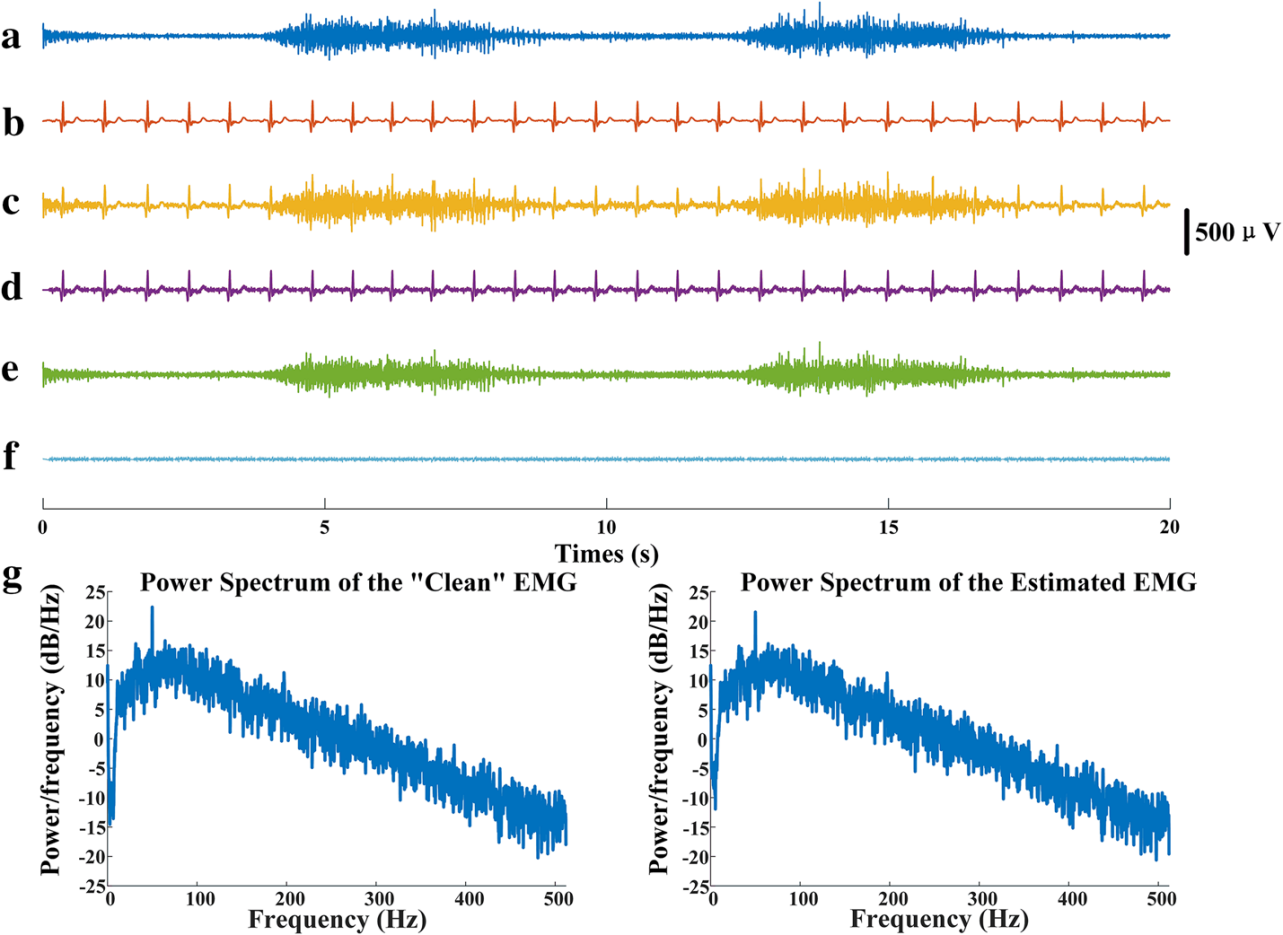
**a** One channel of the “clean” EMG (*lateral movement*, channel 90); **b** the same channel of the “pure” ECG interference; **c** synthetic EMG signal by adding **a** and **b**; **d** the estimated ECG interference; **e** the estimated EMG signal; **f** the residual signal between the “clean” EMG and the estimated EMG; **g** the spectrum of the “clean” EMG (*left*) and the spectrum of the estimated EMG (*right*)

Figure 1 shows an example of ECG removal on synthetic EMG signal. One specific channel of synthetic contaminated EMG signal in Fig. 1c is composed by the “clean” EMG (Fig. 1a) and “pure” ECG (Fig. 1b). After the denoising processing, the estimated ECG, the filtered EMG, and the residual between the “clean” EMG and the filtered EMG are shown in Fig. 1d–f, respectively. Figure 1g presents the power spectrum comparison of the “clean” EMG and the filtered EMG signal after the denoising. It was observed that the FastICA based peel off method imposes little distortion to the EMG component in both amplitude and frequency.

Table 1 reports results for quantitative evaluation of ECG interference removal performance tested on synthetic surface EMG signals. As can be seen, different channels of synthetic ECG contaminated EMG signals had a large range of variation for each of all the three indices before the denoising processing, which can be used to evaluate the performance of the proposed ECG removal method under different interference levels. After the processing, the ECG interferences were separated and then subtracted from synthetic contaminated EMG signals. The significantly increased SIR values and CORR values (approximating to 1), with a reduced range of variations, illustrate the good performance of ECG interference removal. The significantly reduced MFVR values (approximating to 0) further demonstrate the proposed method has little damage to the frequency of EMG.

**Table 1:** A summary of ECG interference removal performance tested on synthetic surface EMG signals

Indices	The synthetic EMG with ECG contamination			The filtered EMG		
	Forward	Backward	Lateral	Forward	Backward	Lateral
SIR (dB)	−3.3 ± 7.7	−0.70 ± 8.1	−0.74 ± 7.9	12.6 ± 2.1	13.4 ± 1.4	12.9 ± 1.2
MVFR (%)	61.4 ± 25.9	63.7 ± 26.5	56.9 ± 27.3	2.7 ± 3.0	1.1 ± 1.3	1.4 ± 1.3
CORR	0.545 ± 0.248	0.620 ± 0.227	0.622 ± 0.228	0.970 ± 0.017	0.976 ± 0.009	0.973 ± 0.009

**Fig. 2:**
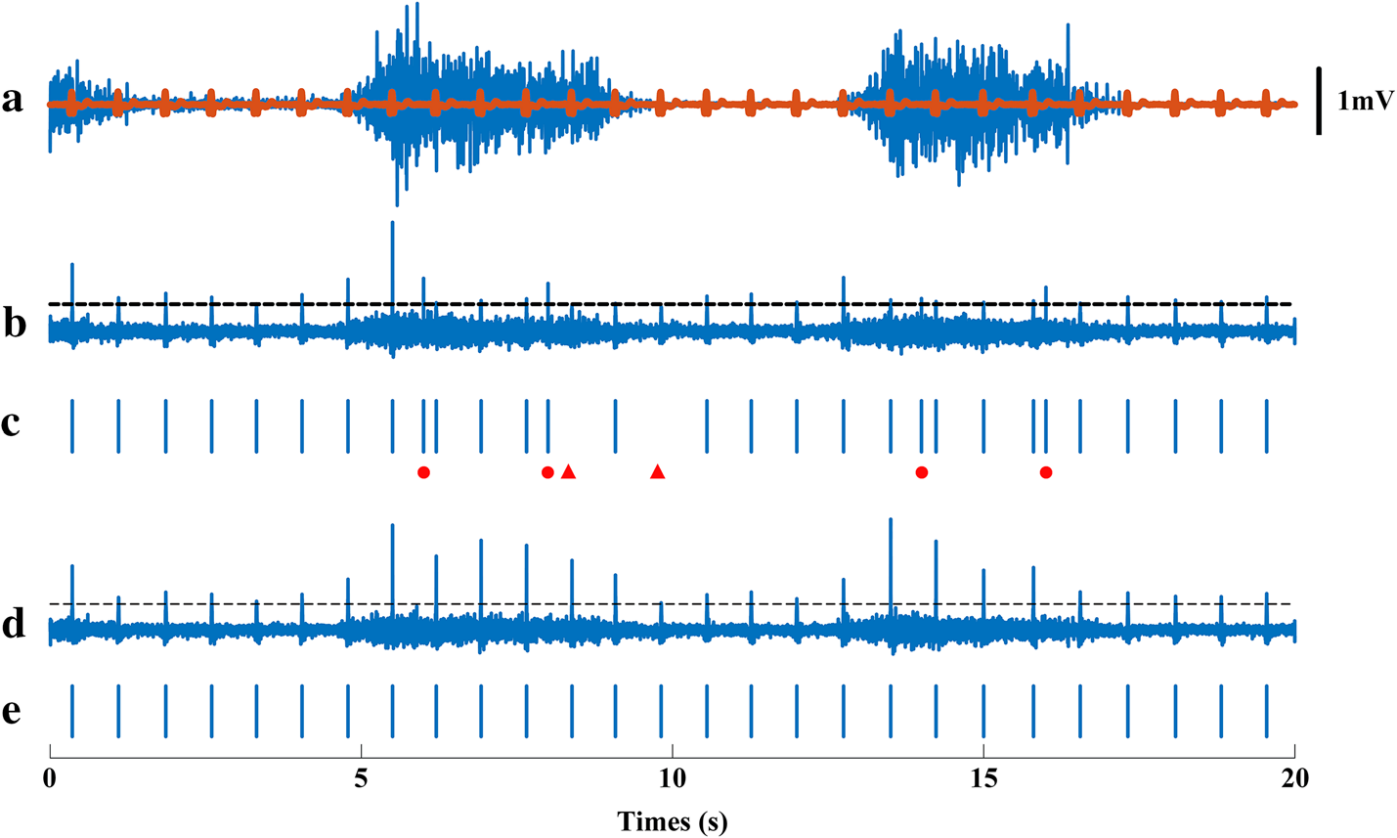
**a** One channel of the contaminated EMG signal (SIR = 13.2). ECG interference in the signal was marked by* bold line*; **b** the output of FastICA; **c** the ECG spike train estimated from **b**, which has false spikes (as indicated by *red circle*) and missing spikes (indicated by *red triangle*); **d** the output of constrained FastICA (using the spike train shown in **c** as a constraint); **e** the ECG spike train estimated from **d**, which is perfectly match the real ECG firing spike train

Figure 2 shows the main process of applying the proposed method to the special testing dataset consisting of a total of 22 channels with relatively large SIRs (SIRs vary from 7.8 to 19 dB). Figure 2a shows one channel of the contaminated EMG signal (SIR = 13.2). ECG interference in the signal is marked by bold line. In this case, FastICA was difficult to converge to the ECG interference with a random initial value. However, the meaningful result was obtained by using the sample at a specific ECG firing instant as the initial value of FastICA, which is shown in Fig. 2b. After applying a threshold on the appropriate FastICA output (Fig. 2b), the ECG spike train was identified (Fig. 2c), but with false spikes (as indicated by red circle) and missing spikes (indicated by red triangle). Figure 2d shows the output of constrained FastICA (using the spike train shown in Fig. 2c as a constraint). With these procedures, the ECG spike train was correctly estimated, as shown in Fig. 2e.

**Fig. 3:**
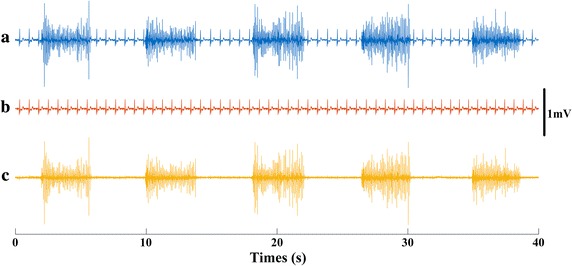
**a** An example of the contaminated EMG (*backward*, channel 108); **b** the estimated ECG interference; **c** the EMG after ECG removal

The proposed method was also tested on real ECG contaminated surface EMG signals. An example of satisfactory performance is shown in Fig. 3.

## Discussion

In this study, we developed a novel FastICA based method to remove ECG interference from multi-channel surface EMG signals. There are three key components or steps in the design. First, FastICA is used to produce a rough estimate of the firing spike train of the ECG interference. Then, constrained FastICA is applied to strengthen the reliability of the estimated ECG spike train. Finally, the ECG interference waveforms in different channels are estimated and subtracted straightforwardly from the contaminated signals.

The proposed method in this study can be viewed as a special case of our recently developed progressive FastICA peel-off (PFP) framework for high density surface EMG decomposition [12]. To overcome the local convergence of FastICA, PFP uses the firing information of the already identified motor units through FastICA to estimate their motor unit action potential waveforms and then subtract them from original EMG signal through a peel-off step. Such a strategy is applied progressively on the residual signal to expand the set of the motor unit spike trains (or to decompose more motor units). PFP has demonstrated much higher decomposition yield as compared with previous ICA applications for high density surface EMG decomposition. In the current study, we similarly treat ECG interference as an independently and continuously firing action potential train and focus on extraction of this particular component.

Compared with EMG or superimposed motor unit action potential trains, ECG interference is more sparse and usually has relatively large amplitude. Therefore, ECG spike train often emerges as the first component identified by FastICA. Furthermore, if the recording during muscle relaxation is available, the observed ECG firing spikes can be used to help set appropriate initial values in FastICA thus facilitating the algorithm’s convergence toward ECG interference in the presence of surface EMG.

Compared with other ICA-based ECG removal methods, the present study provides a new perspective to solve the problem. For example, both [9] and [11] simply regarded the output of FastICA as proportional to ECG interference. Then the “ECG interference” was used as a reference signal of an adaptive filter [11] or directly subtracted from the original ECG contaminated signal [9]. Similarly, the outputs of ICA without ECG interference were simply viewed as the denoised EMG signals, which are actually different from the original EMG components [15]. As mentioned earlier, such applications assume that the waveforms across multiple channels are consistent (e.g. ECG waveforms in [9, 11]; both ECG and EMG waveforms in [15]). Unfortunately, such an assumption is not always true due to different volume conductor effects between the signal source and the recording channels. By contrast, the FastICA applied in our method acted as a tool for detecting and pre-estimating an ECG spike train (which is consistent across channels), whereas their corresponding ECG waveforms across the output independent components are probably distorted through the FastICA filtering and therefore discarded. Based on the instants of ECG spike train further identified by subsequent constrained ICA, the true waveforms of ECG in different channels can be estimated accurately and then subtracted straightforwardly. Note that in [9], the temporally constrained ICA technique was also used to facilitate estimating ECG artifacts, which required to incorporate the priori information of all the possible firing instants of ECG. However, this priori information is not always accessible when processing EMG signals with less significant ECG contamination (such as the signal demonstrated in Fig. 2). In our study, this information can be roughly (usually precisely in practice) pre-estimated by the FastICA filtering first, and then the constrained FastICA is performed to further improve the precision of the ECG firing spike train estimation.

Thus, a primary feature of the developed method is the two-step combination of the FastICA and the constrained FastICA in order to automatically and reliably estimate the ECG spike train directly from the original multichannel surface EMG recordings, which allows improved performance of ECG interference removal even over a wide range of ECG contamination levels. The performance of the developed method was tested with both synthetic and experimental surface EMG signals contaminated by different levels of ECG interference, and satisfactory performance was actually demonstrated.

It is also noteworthy that the proposed method requires to work on multiple channels of surface EMG signals, specifically suitable for filtering high-density surface EMG array data. However, since implementation of both the ICA approach and the “peel-off” step need a bulk of data with sufficient time duration, the proposed method is recommended for offline data processing. By temporally dividing a signal stream into consecutive data blocks (each with a time duration of at least a few seconds), the proposed method may also be used in some time-insensitive circumstances.

## Conclusions

A novel FastICA-based peel-off method for ECG interference removal from multi-channel surface EMG signals is proposed in this study. In the proposed method, a two-step FastICA processing is employed to accurately estimate a train of ECG firing instants, which is used for subsequent ECG waveform estimation and separation from individual EMG channels. It has been demonstrated that the proposed method is able to remove ECG interference efficiently with little distortion to surface EMG in both amplitude and frequency, thus offering a new and practical tool for filtering multi-channel EMG signals to eliminate ECG interference.
